# Exploring Appropriate Positioning of the Spiral Blade in Treatment of Subtrochanteric Fractures of the Femur Using Proximal Femoral Nail Antirotation

**DOI:** 10.1111/os.70051

**Published:** 2025-06-17

**Authors:** Qingyan Zhang, Xiaogang Wang, Longhui Su, Qiang Xu

**Affiliations:** ^1^ Department of Lower Limbs Sichuan Orthopaedic Hospital Chengdu China

**Keywords:** calcar‐referenced tip‐apex distance (Cal‐TAD), proximal femoral nail antirotation (PFNA), subtrochanteric fractures, tip‐apex distance (TAD)

## Abstract

**Objectives:**

Subtrochanteric fractures have anatomic characteristics distinct from intertrochanteric fractures that may affect the positioning of the spiral blade during surgical treatment. Tip‐apex distance (TAD) and calcar‐referenced tip‐apex distance (Cal‐TAD) were measured to determine if these measures are reliable indicators to assist in the accurate placement of intramedullary nails and minimize postoperative complications.

**Methods:**

For patients treated with proximal femoral nail antirotation (PFNA) internal fixation between 2016 and 2020, we analyzed the TAD, Cal‐TAD, and postoperative complications. Fracture healing was assessed radiographically at 6‐week intervals until union. The incidences of axial cut‐off, cephalad cut‐off, and non‐union were also examined. Analysis of variance and Fisher's exact test were performed to evaluate differences in complications between the TAD and Cal‐TAD groups.

**Results:**

Data from 104 patients (58 males, 46 females) with a mean age of 56.9 years were analyzed. Fracture healing was observed in 90 (86.5%) patients at an average time of 14.92 ± 1.81 weeks. The healing rate was significantly higher when the TAD and Cal‐TAD were controlled within the 20–25 mm range (*p* < 0.05). Postoperative complications occurred in 14 (13.5%) cases [cephalad cut‐off, *n* = 5 (4.8%); axial cut‐off, *n* = 4 (3.8%); non‐union, *n* = 5 (4.8%)]. Five (4.8%) complications occurred without internal fixation failure. The fracture healing time and incidence of complications differed among groups defined by TAD and Cal‐TAD measurements, and were shortest and lowest, respectively, in the 20 mm < TAD/Cal‐TAD < 25 mm group.

**Conclusions:**

In our cohort, use of PFNA internal fixation for treatment of unstable femoral subtrochanteric fractures and placement of the spiral blade in the middle or lower 1/3 of the femoral neck did not increase the incidence of complications. Therefore, we propose that the TAD rule of 20–30 mm should not apply to subtrochanteric fractures, and TAD and Cal‐TAD should be controlled within the range of 20–25 mm to reduce the incidence of complications.

## Graphical abstract

Given the differences in age, injury mechanism, and anatomy between subrotator and intertrochanteric fractures, the conventional TAD and CAL‐TAD may not be suitable for intertrochanteric fractures. This study retrospectively reviewed subrotator fractures complications to find the optimal position for subrotator fractures.

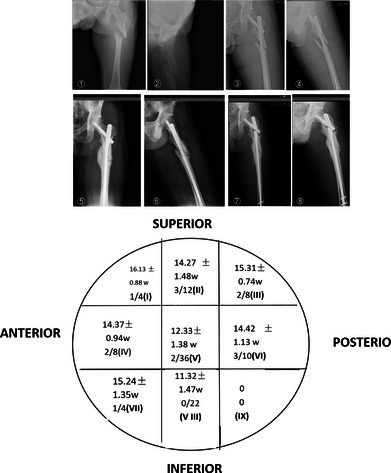

**Abbreviations**
Cal‐TAD: calcar‐referenced tip‐apex distance
DR: repeat digital radiography
IM: intramedullary
PFNA
: proximal femoral nail antirotation
TAD: tip‐apex distance

## 1 Introduction

Subtrochanteric fractures account for 10%–34% of all proximal femoral fractures and often occur within 5 cm of the lesser trochanter and its distal end [1, 2]. Some scholars have proposed that subtrochanteric fractures can be located from the lesser trochanter to the middle and upper third of the proximal femur [3]. Due to the biomechanical and anatomic characteristics of the subtrochanteric femur, a concentration of stress occurs in this region [4]. Thus, conservative treatment of fractures in this area can lead to fracture malunion, non‐union, or other complications. Therefore, surgery is often the first‐line treatment for subtrochanteric fractures [5], with either extramedullary external fixation represented by sliding hip screws (SHS) or intramedullary (IM) represented by proximal femoral nail antirotation (PFNA) [6]. Generally, PFNA is preferred due to its advantages involving fixation rigidity, surgical injury, and fracture healing [7]. Electing for PFNA can also shorten both operation time and duration of hospital stay, reduce intraoperative bleeding, and is less invasive [8]. However, subtrochanteric fractures pose unique challenges due to their distinct anatomic and biomechanical properties, which often result in suboptimal surgical outcomes. The incidence of non‐union, implant failure, refracture, and other postoperative complications in these cases is 19%–32% non‐union, and second surgical interventions are necessary in some cases [9]. Additionally, Vélez et al. [10] documented a failure rate of 6%–21% for the internal fixation of hip fractures using IM nails. This procedure also increases the susceptibility to specific complications, including the cutting out of the spiral blade and the need for nail removal. The tip‐apex distance (TAD) has been found to be an important indicator of postoperative failure of the treatment of subtrochanteric fractures with IM nails. TAD was first proposed by Baumgertner in 1995, and it refers to the distance measured from the tip of the lag screw to the apex of the femoral head on anteroposterior and lateral radiographs [11]. This distance is relevant for optimal placement of the blade for intramedullary devices. Currently, there is no standardized distance that is recommended. However, measurements of the TAD and calcar‐referenced tip‐apex distance (Cal‐TAD) on anteroposterior and lateral radiographs of subtrochanteric fractures have indicated that these distances should be < 25 mm to reduce complications ofcut‐out and nail breakage, fracturenon‐union, and poor functional outcomes [12].

It has been proposed that a TAD < 25 mm can effectively prevent the spiral blade from cutting off the femoral head. However, others have proposed a range of 20–30 mm is needed [13, 14]. To date, data regarding optimal placement of the blade of intramedullary devices is limited, and optimal placement for intramedullary devices such as PFNA is lacking. Furthermore, most of the literature available derives from SHS, and it remains inconclusive whether these results are also applicable to PFNA. It has been reported that screw deviation during internal fixation leads to a TAD > 25 mm, yet greater fracture stability is achieved [12]. In 2012, Kuzyk et al. proposed the concept of “calcar‐referenced tip‐apex distance”, or Cal‐TAD [15]. Kuzyk advocated that the femoral neck screw should be as close to the femoral calcar as possible during internal fixation, and this is consistent with the anatomic and biomechanical characteristics of the proximal femur. However, the optimal range for Cal‐TAD remains uncertain.

This study was conducted to determine (i) whether the TAD and Cal‐TAD are reliable indicators of accurate IM nail placement in the treatment of subtrochanteric femoral fractures and (ii) whether the control of the TAD and Cal‐TAD within specific ranges can minimize postoperative complications. Specifically, we sought to challenge the conventional TAD range of 20–30 mm and propose a narrower, evidence‐based range that could reduce the incidence of complications such as blade cut‐out and nonunion in patients treated with PFNA internal fixation.

## 2 Materials and Methods

The Ethics Committee of our hospital approved this study (no. KKY2021‐026‐01). All procedures were conducted in compliance with ethical standards.

### 2.1 Patient Demographics

We retrospectively analyzed 142 patients who were diagnosed with a subtrochanteric fracture and underwent PFNA surgery at our hospital between January 2016 and January 2020. The patients were contacted by phone to explain the purpose, methods, data to be collected, and potential risks of this study. It was also clearly emphasized that participation was voluntary. Personal data were maintained in compliance with applicable privacy regulations, and patients' identities and personal information were protected. Measures during follow‐up, including multiple requests for outpatient follow‐up and repeat digital radiography (DR) examinations to monitor the occurrence of complications, were also explained to the participants. Written informed consent was obtained from the patients and/or their families to allow data to be submitted for publication, and this study was approved by the Ethics Committee of our hospital.

All of the surgeries performed were completed by the same group of trauma orthopedic doctors and included PFNA internal fixation. Inclusion criteria were: (1) age ≥ 18 years; (2) a clear history of trauma; (3) fracture of the subtrochanteric femur diagnosed by x‐ray and computed tomography; (4) PFNA internal fixation was elected and all fractures achieved satisfactory reduction; (5) a standardized rehabilitation regimen was performed after surgery; and (6) patient follow‐up was continued for at least 12 months, and complications were recorded. Patients were excluded if: (1) multiple trauma occurred and other fractures were present in the ipsilateral limb; (2) previous hemiplegia or muscle strength decline was caused by various reasons; (3) significant obstacles to cardiopulmonary function exist which affect liver and/or kidney function to prevent surgery; (4) serious blood system diseases were present; (5) old fractures or pathological fractures were present; or (6) history of long‐term use of bisphosphonates.

### 2.2 Surgical Procedures

All patients underwent surgery within 3 days after their trauma event. The surgical position was supine, and all of the patients preferred closed reduction. If the latter became difficult, limited open reduction through the lateral submuscular approach was adopted. The healthy lower limb was flexed and fixed in the abduction position to perform C‐arm x‐ray fluoroscopy during surgery. The injured limb was properly lifted and abducted to achieve ~30° traction. Closed reduction of fractures was achieved by adjusting traction force, limb alignment, and rotation. A C‐arm x‐ray image of the affected hip joint in the frontal and lateral positions was obtained to check reduction quality. After achieving satisfactory fracture reduction, a longitudinal incision ~3.0–4.0 cm long from the tip of the femoral trochanter to the proximal end of the affected side was made. The apex of the greater trochanter was explored to select an entry point for the guide. A guide wire was then inserted through the fracture line up to the distal femur. After checking that the guide wire was in position, the femur drill was reamed to insert the guide needle into the medullary cavity. Positioning of the guide needle in the middle of the medullary cavity was confirmed with positive and lateral fluoroscopy performed by a C‐arm x‐ray machine. Next, the femur was reamed, an appropriate intramedullary nail was selected for insertion into an appropriate length, locking screws were placed into the nail, and fracture reduction and internal fixation positions were rechecked. The guide needle was drilled through the femoral neck to ensure that the guide needle was located in the center or at 1/3 of the middle and lower region of the femoral neck. The lateral position was located in the center of the femoral neck. After sounding was performed, a spiral blade of appropriate length was inserted and locked, followed by the insertion of two locking nails under distal fluoroscopy. Finally, the proximal end was screwed into the tail cap. In some cases, limited incisions can be used for fracture reduction procedures. In such cases, the operator makes a small incision on the lateral thigh near the fracture to expose the fracture end and then uses standard instruments (e.g., top rod, reduction forceps, etc.) to achieve reduction and temporary fixation. The reduction is stabilized with intramedullary implant nails, locking the proximal end, and then the fracture end is bound and fixed with titanium cables.

### 2.3 Postoperative Recovery

First generation cephalosporin antibiotics were administered intravenously 24 h postoperatively as perioperative prophylaxis. The following standardized rehabilitation regimen was applied after surgery. Immediately following surgery, the patients began ankle flexion, extension, and rotation exercises. On the first day after surgery, hip and knee flexion and extension exercises and quadriceps femoris contraction training were introduced. This early mobilization helped to prevent complications such as deep vein thrombosis and stiffness. Under the direct supervision of the medical staff, the patients also started walking with crutches while avoiding weight bearing on the affected limb. This non‐weight‐bearing ambulation was maintained for the initial phase of recovery. At 4–6 weeks postoperatively, when radiographic evidence of bone callus formation indicating fracture healing was observed, weight‐bearing exercises for the affected limb were introduced gradually. This transition was managed carefully, with the amount of weight bearing increased incrementally based on each patient's progress and comfort level, as assessed during regular follow‐up visits.

### 2.4 Radiological Assessment

Bone mineral density (BMD) was measured pre‐operatively using a GE Lunar dual‐energy X‐ray absorptiometry (DXA) scanner (Model: iDXA). Immediate postoperative radiographs, when available, were analyzed for blade tip position according to the Cleveland zone [16], TAD, and Cal‐TAD. Calculation of TAD has previously been reported in detail. [11] Briefly, it is represented by the distance measured from the tip of the lag screw to the apex of the femoral head on anteroposterior (AP) and lateral radiographs. We adjusted for radiographic magnification using the hip screw as a known diameter [17]. Cal‐TAD is the sum of TAD in the lateral view and the distance between a line tangent to the medial cortex of the femoral neck and the tip of the lag screw in the AP view [18].

TAD ranged between 16 and 32 mm (mean: 23.3 ± 0.7 mm). Previous literature has reported appropriate TAD values for the treatment of intertrochanteric fractures using PFNA ranging from 20 mm to 30 mm [19]. Therefore, we established the following groups with TAD as the group index: group A (TAD ≤ 20 mm, *n* = 30), group B (20 mm < TAD < 25 mm, *n* = 41), and group C (TAD ≥ 25 mm, *n* = 33). When Cal‐TAD was used as the group index, the groups included: A_1_ (Cal‐TAD ≤ 20 mm, *n* = 22), group B_1_ (20 mm < Cal‐TAD < 25 mm, *n* = 61), and group C_1_ (Cal‐TAD ≥ 25 mm, *n* = 21).

Patients were re‐evaluated at 6 weeks, 3 months, 4.5, 6, 9, and 12 months postoperatively until radiological union occurred. The time required for fracture healing was recorded. To eliminate interobserver variability, the chief researcher evaluated fracture reduction according to the criteria of Baumgaertner et al. [17], classifying it as anatomic reduction (fracture fragments returned perfectly to their normal anatomic positions, with no visible displacement or rotation visible on radiographs) or near‐anatomic reduction (fracture fragments close to their normal anatomic positions, with minimal displacement or rotation that does not significantly affect the overall alignment or healing outcome). Cases that lacked restoration to the normal anatomic axis and exhibited separation of the fracture fragments from each other were classified as non‐anatomic reductions.

### 2.5 Complications

Complications of fracture healing and internal fixation were recorded. The former included fracture non‐union and delayed union. Non‐union is clinically defined as a lack of cortical bridging in at least three cortices 9 months post‐surgery. For internal fixation, cut‐out cases were counted according to Aicale et al. [12] Briefly, they were defined as an increase of TAD or Cal‐TAD distance by more than 3 mm due to abnormal movement of the cephalic screw or nail after surgery. This included cephalad cut‐out (versus head collapse) and axial cut‐out (medial or anterosuperior migration). To avoid repeated statistics, patients with delayed union and non‐union caused by internal fixation‐related complications were included in the group of internal fixation complications. Only the total number of complications without specific distinction was counted.

### 2.6 Statistical Analysis

Data were analyzed using SPSS statistical software version 22.0 (SPSS Inc. Chicago, IL, USA). Analysis of variance (ANOVA) was used to evaluate statistically significant differences in complications between the TAD and Cal‐TAD groups. As 20% of the contingency tables had expected counts of < 5, we employed Fisher's exact test to evaluate differences in categorical variables in accordance with the Cleveland system.

## 3 Results

### 3.1 Patient Demographic and Clinical Features

A total of 142 patients met inclusion criteria. However, 38 patients were lost to follow‐up. For two patients, an x‐ray examination conducted 3–6 months after surgery showed union of the fractures and a return to normal walking was achieved. However, these cases were excluded due to deaths caused by cardiovascular and cerebrovascular diseases. The remaining 36 cases did not include any complications such as loosening of internal fixation, cutting out, or hip varus deformation. They were excluded because they did not include at least 12 months of follow‐up. Thus, data from 104 patients (58 males, 46 females) with a mean age of 56.9 (range, 24–76) years were analyzed. Fifty cases involved left–sided fractures and 54 cases involved right‐sided fractures. Causes of injury included a tumble (*n* = 45), a fall from height (*n* = 24), and traffic accidents (*n* = 35). According to AO/OTA classification, the sample comprised 44 (42.3%) class A, 46 (44.3%) class B, and 14 (13.4%) class C cases. DXA disclosed normal BMD (T ≥ −1.0) in 15 patients, osteopenia (−2.5 < T < −1.0) in 32 patients, and osteoporosis (T ≤ −2.5) in 57 patients.

### 3.2 Implant Positioning

Long intramedullary nails (> 240 mm) were selected for all internal fixation procedures. Static locking was selected for distal locking. Fracture reductions were evaluated by the chief researcher to eliminate inter‐observer variability and were classified aseither anatomic, near‐anatomic, or non‐anatomic types. In total, 80 (76.9%) cases were classified as anatomic reductions, 22 (21.2%) cases were classified as near‐anatomic reductions, and 2 (1.9%) cases were classified as non‐anatomic reductions.

### 3.3 Postoperative Complications

At a mean follow‐up duration of 13 months, fracture healing was observed in 90 (86.5%) patients. The average time to healing was 14.92 ± 1.81 weeks. The healing rate was significantly higher in cases in which the TAD (40/41, 97.6%) and Cal‐TAD (59/61, 96.7%) were controlled within the 20–25 mm range (*p* < 0.05). Postoperative complications occurred in 14 (13.5%) cases; 5 (4.8%) cases were pure non‐union without internal fixation failure, and the remaining 9 cases [4 (3.8%) axial cut‐offs and 5 (4.8%) cephalad cut‐offs] involved internal fixation failure (Table 1).

**TABLE 1:** Complications reported.

No.	Age (years)	M/F	Cause of injury	AO/OTA class	Cleveland system	Quality of reduction	TAD	Cal‐TAD	Complication^a^
1	72	M	Tumble	32 A2.3	Centro‐central	Anatomic	17.1	19.4	Axial cut‐out
2	64	F	Tumble	32 A2.1	Centro‐central	Anatomic	16.9	19.1	Axial cut‐out
3	56	F	Tumble	32 B2.1	Centro‐central	Anatomic	15.4	17.2	Non union
4	63	F	Traffic accident	32 A3.3	Anterior‐central	Anatomic	11.3	16.7	Axial cut‐out
5	62	M	Tumble	32 C2.1	Centro‐central	Near‐anatomic	22.3	23.4	Non union
6	67	F	Traffic accident	32 B2.3	Infero‐central	Anatomic	26.7	24.5	Axial cut‐out
7	57	F	Fall from height	32 A2.1	Posterior‐central	Near‐anatomic	34.2	36.2	Cephalad cut‐out
8	61	M	Traffic accident	32 A3.3	Centro‐central	Near‐anatomic	32.3	33.7	Non union
9	67	M	Traffic accident	32 C2.1	Infero‐central	Near‐anatomic	29.4	31.2	Cephalad cut‐out
10	64	F	Traffic accident	32 B2.2	Infero‐central	Non‐anatomic	27.5	31.4	Non union
11	55	F	Tumble	32 B2.2	Centro‐central	Non‐anatomic	28.9	29.7	Non union
12	55	M	Tumble	32 A3.3	Posterior‐central	Near‐anatomic	36.4	34.5	Cephalad cut‐out
13	60	F	Traffic accident	32 C2.2	Posterior‐central	Anatomic	33.2	31.2	Cephalad cut‐out
14	47	M	Fall from height	32 C2.3	Infero‐central	Near‐anatomic	37.7	38.5	Cephalad cut‐out

^a^ Axial cut‐out complication due to blade migrating superiorly; cephalad cut‐out complication due to blade migrating superiorly.

**FIGURE 1:**
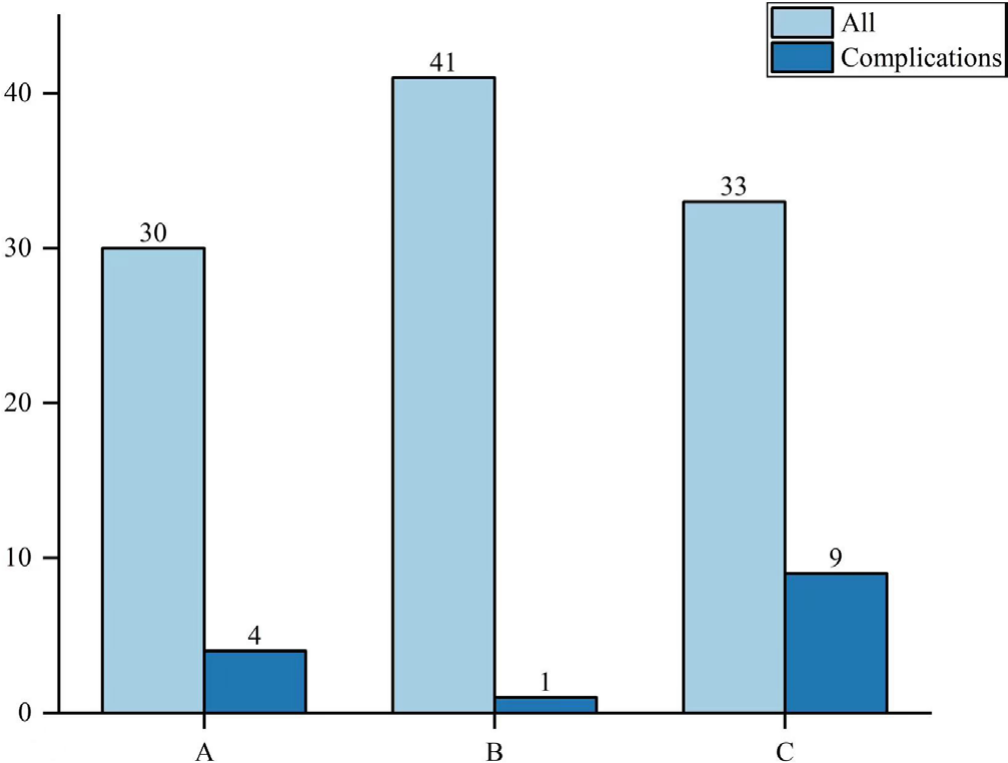
Complications according to TAD (mm).

**FIGURE 2:**
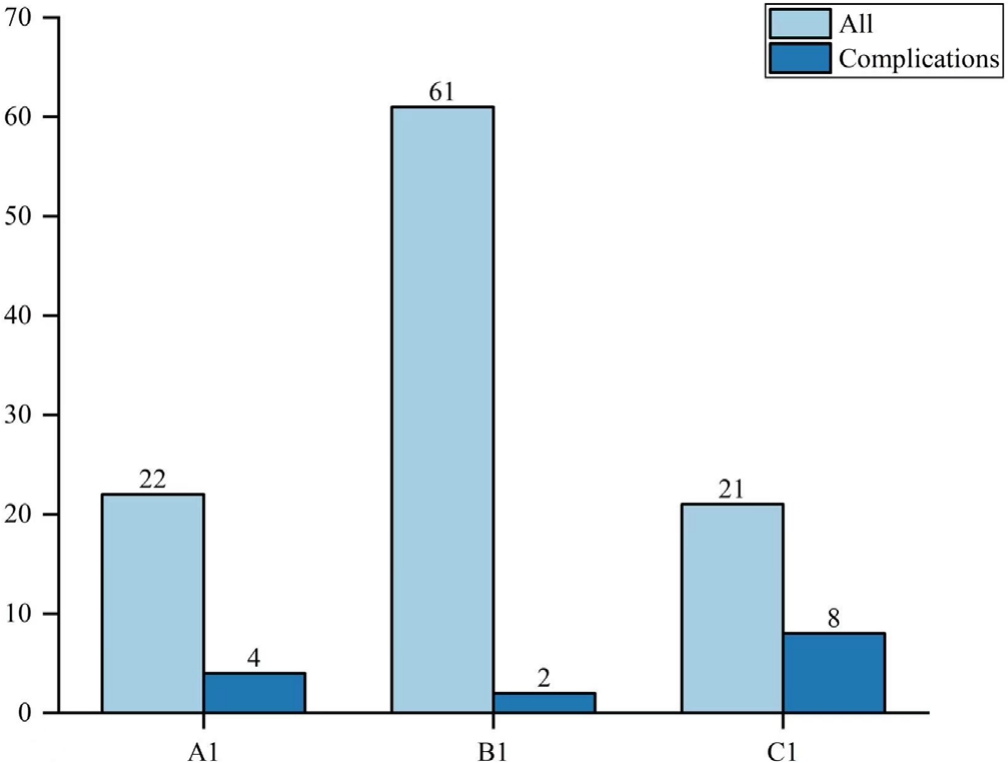
Complications according to Cal‐TAD (mm).

Baseline characteristics (sex, age, and mode of injury) did not differ significantly among TAD groups A–C (Table 2, Table 3). The healing time was shorter in group B (13.41 ± 1.57 weeks) than in groups A (15.92 ± 1.74 weeks) and C (15.63 ± 1.85 weeks; *p* = 0.027). In addition, significantly fewer complications occurred in group B than in groups A and C (*p* = 0.004, Figure 1). Similarly, the baseline data did not differ significantly among Cal‐TAD groups A1–C1. The healing time was shorter in group B1 (13.37 ± 1.43 weeks) than in groups A1 (15.48 ± 1.88 weeks) and C1 (14.67 ± 1.73 weeks; *p* = 0.003). The incidence of complications was lower in group B1 than in groups A1 and C1 (*p* = 0.001 Figure 2).

**TABLE 2:** Comparison of baseline TAD data.

Variables	Group A (*n* = 30)	Group B (*n* = 41)	Group C (*n* = 33)	*p*
Age (years)				
Mean	51.3 ± 12.1	50.2 ± 10.7	52.6 ± 11.3	0.786
Range	24–68	25–76	26–69	
Gender, *n* (%)				0.677
Female	13 (43.3)	20 (48.8)	13 (39.4)	
Male	17 (56.7)	21 (51.2)	20 (60.6)	
Side, *n* (%)				0.754
Left	13 (53.3)	20 (56.1)	17 (51.5)	
Right	17 (46.7)	21 (43.9)	16 (48.5)	
AO/OTA classification, *n* (%)				0.058
32 A	12 (40.0)	14 (34.1)	18 (54.5)	
32 B	14 (46.7)	20 (48.8)	12 (36.4)	
32 C	4 (13.3)	7 (17.1)	3 (9.1)	
Causes of injury, *n* (%)				0.411
Tumbles	15 (50.0)	15 (36.6)	15 (45.5)	
Falls from height	5 (16.7)	13 (31.7)	6 (18.2)	
Traffic accidents	10 (33.3)	13 (31.7)	12 (36.3)	
Reduction quality, *n* (%)				0.714
Anatomic	24 (80.0)	32 (78.0)	24 (72.7)	
Near‐anatomic	6 (20.0)	8 (19.5)	8 (24.2)	
Non‐anatomic	0 (0)	1 (2.5)	1 (3.1)	
Bone mineral density, *n* (%)				0.713
Normal bone mass (*T* ≥ −1.0)	4 (13.3)	6 (14.6)	5 (15.2)	
Osteopenia (−2.5 < T < −1.0)	11 (36.7)	11 (41.5)	10 (42.4)	
Osteoporosis (T ≤ −2.5)	15 (50.0)	24 (43.9)	18 (42.4)	

**TABLE 3:** Comparison of baseline data of Cal‐TAD.

Variables	Group A_1_ (*n* = 22)	Group B_1_ (*n* = 61)	Group C_1_ (*n* = 21)	*p*
Age (years)				
Mean	49.1 ± 11.1	56.2 ± 17.4	55.1 ± 16.3	0.081
Range	24–67	27–76	31–70	
Gender, *n* (%)				0.073
Female	9 (43.3)	25 (41.0)	12 (39.4)	
Male	13 (56.7)	36 (59.0)	9 (60.6)	
Side, *n* (%)				0.316
Left	10 (45.5)	30 (49.2)	10 (47.6)	
Right	12 (54.5)	31 (50.8)	11 (52.4)	
AO/OTA classification, *n* (%)				0.517
32 A	8 (36.4)	26 (42.6)	10 (47.6)	
32 B	10 (45.5)	29 (47.5)	7 (33.3)	
32 C	4 (18.2)	6 (9.9)	4 (9.1)	
Causes of injury, *n* (%)				0.532
Tumbles	11 (50.0)	22 (36.1)	12 (57.1)	
Falls from height	2 (9.1)	18 (29.5)	4 (19.0)	
Traffic accident	9 (40.9)	21 (34.4)	5 (23.8)	
Reduction quality, *n* (%)				0.373
Anatomic	17 (77.3%)	48 (78.7)	15 (71.4)	
Near‐anatomic	5 (22.7%)	12 (19.7)	5 (23.8)	
Non‐anatomic	0 (0)	1 (1.6)	1 (4.8)	
Bone mineral density, *n* (%)				0.544
Normal (*T* ≥ −1.0)	3 (13.3)	8 (14.6)	4 (15.2)	
Osteopenia (−2.5 < *T* < −1.0)	9 (36.7)	15 (41.5)	8 (42.4)	
Osteoporosis (*T* ≤ −2.5)	10 (50.0)	38 (43.9)	9 (42.4)	

**FIGURE 3:**
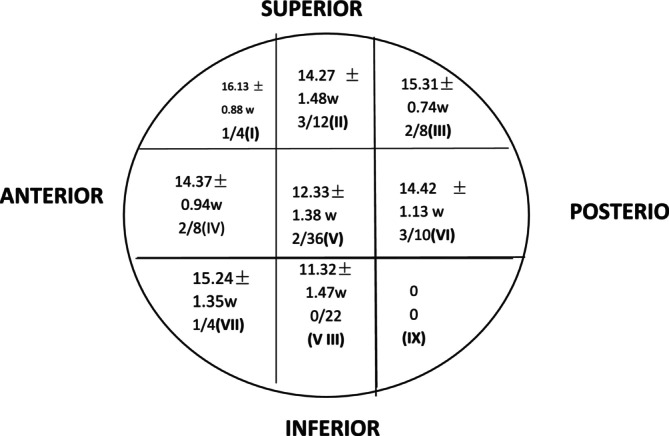
The distribution of lag screw positions in the femoral head and numbers of complications for each position.

**FIGURE 4:**
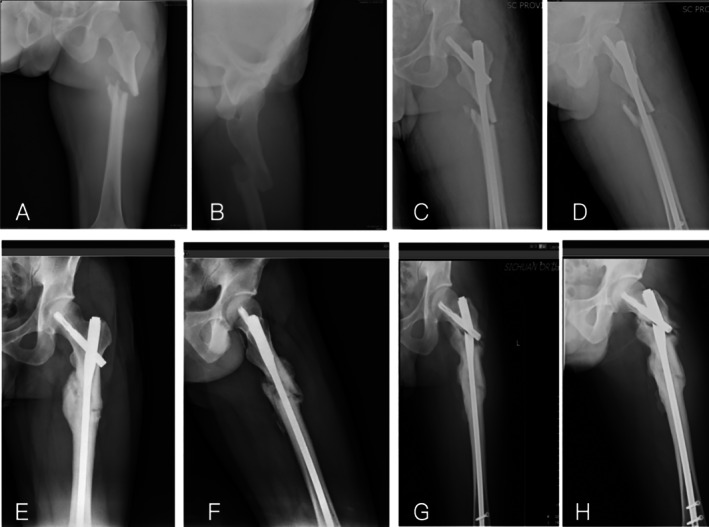
A 67‐year‐old male truck driver was hospitalized following a car accident. The preoperative DX film is shown in (A, B). The patient underwent extended PFNA surgery 2 days after the surgery (C, D). The immediate postoperative TAD value was 29.4 and the Cal‐TAD value was 31.2. Postoperative follow‐up at 9 months revealed delayed union of the fracture (E, F). At 12 months after surgery, the patient experienced left hip pain while walking. DX examination revealed non‐union of the fracture with internal fixation failure (G, H).

**FIGURE 5:**
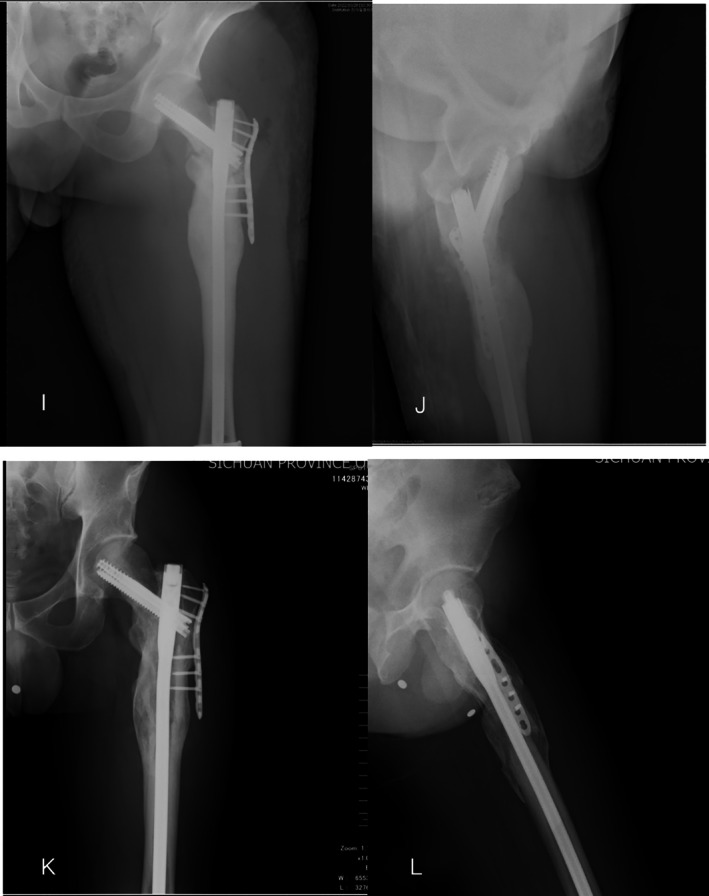
Images obtained during immediate postoperative DX examination (I, J). Repeat DX examination performed 14 weeks postoperatively (K, L) showed that the fracture had healed well.

The shortest healing time was observed in Cleveland zone VIII (11.32 ± 1.47 weeks), followed by zone V (12.32 ± 1.38 weeks). Complications occurred most frequently in zone VI (middle–posterior; 33.3%). No complications occurred in zone VIII (middle–inferior; 0%), and two complications occurred in zone V (middle–middle; 5.6%; *χ* ^2^ = 11.816, *p* = 0.015; Figure 3). Typical cases are shown in Figures 4 and 5.

### 3.4 Management of Spiral Blade Cut‐Out

The main internal fixation–related complications were axial and cephalad cut‐out. Among cases with TADs < 20 mm, 60% of complications involved axial cut‐out; among those with TADs > 25 mm, ~55% of complications involved cephalad cut‐out. These complications were addressed with a comprehensive treatment strategy including the reassessment and stabilization of the fracture site, the management of cut‐out with open reduction and supplementary fixation (e.g., cerclage wiring), and the potential use of bone grafts to promote healing. In complex cases involving femoral head and subtrochanteric lesions, advanced augmentation techniques including the use of polymethylmethacrylate or off‐label use of devices such as reconstruction nails were considered.

Revision surgeries involved the replacement of the internal fixation elements, refreshing of the fracture ends via the removal of ineffective bone callus, and performance of autologous iliac bone grafting (Figure 5). The removal of a broken primary nail can be challenging. When necessary, the fracture was exposed, the proximal broken part was removed, a guide pin was inserted, and a femoral retrograde IM nail was used to remove the remaining fixation.

Given the occurrence of significant bone loss after initial PFNA surgery, Intertan + Lateral locking plates were used with thicker nails for enhanced stability. During surgery, the fractured ends were refreshed, and autologous iliac bone grafting was performed. We generally avoided the removal of the internal fixation after healing to minimize the refracture risk.

## 4 Discussion

### 4.1 Main Findings

In the present clinical study, statistically significant differences in the incidence of complications between patients in groups B and B_1_, and in patients in groups A, C, A_1_, and C_1_, respectively, were observed. These results indicate that TAD and Cal‐TAD can provide important guidance for internal PFNA in the treatment of femoral subtrochanteric fractures. They may also have predictive values for the occurrence of postoperative complications. Given the statistical association of TAD and Cal‐TAD values within the 20–25 mm range with a reduced incidence of complications in the present cohort, we propose the narrowing of the recommended TAD range of 20–30 mm for subtrochanteric fractures to 20–25 mm. We examined 9 (8.7%) cases of internal fixation‐related complications among 104 cases of subtrochanteric fractures. The main manifestations of spiral blade cut‐out were axial cut‐out and cephalad cut‐out. When the TAD was < 20 mm, 60% of the complications reported involved axial cut‐out of the spiral blade. When the TAD was > 25 mm, ~55% of the complications involved cephalad cut‐out. The same results were obtained when Cal‐TAD was < 20 mm and Cal‐TAD was > 25 mm, respectively. Thus, too short a TAD and Cal‐TAD can cause axial cut‐out of the spiral blade, while too long a TAD and Cal‐TAD can cause cephalad cut‐out of the spiral blade.

### 4.2 Background and Clinical Implications of the TAD and Cal‐TAD

Subtrochanteric fractures differ from intertrochanteric fractures in many aspects. First, the age demographics of patients with subtrochanteric fractures are bimodal. Subtrochanteric fractures are often caused by low‐energy injuries in elderly patients but mostly due to high‐energy injuries such as car accidents in young patients [20]. Bone quality and trauma mechanism also differ between these two age groups. As a result, the choice of treatment methods and postoperative effects vary. In the present study, the average age of our cohort members was 56.9 years, which is relatively young. In addition, most of the cases involved high‐energy injuries such as high falling injury and car accidents (*n* = 59, 56.7%). In contrast, intertrochanteric fractures most often involve low‐energy injuries such as falling among the elderly. A second key difference is that a subtrochanteric fracture is located in the cortical bone region, and the contact surface of the fracture end is smaller than that of an intertrochanteric fracture. Muscular attachments such as hip absorbers, adductors, short external rotators, and iliopsoas greatly impact the cortical bone region, thereby resulting in abduction, external rotation, flexion displacement of the proximal fracture block, and inward and upward displacement of the distal end [20]. Therefore, compared with an intertrochanteric fracture, intraoperative reduction is more difficult. In addition, the subtrochanteric region of the femur has a high concentration of stress applied to it, making it susceptible to comminuted fractures. Anatomically, this area is composed of hard cortical bone which is associated with long fracture healing times and a 20% failure rate of internal fixation [21]. Thus, it is consistent that the most suitable position for the spiral blade to treat a subtrochanteric fracture may differ from that for an intertrochanteric fracture.

To date, optimal values for TAD and Cal‐TAD in PFNA of subtrochanteric fractures have not been reported. In 2004, Pervez et al. proposed that TAD is the most important factor in predicting lag screw cutting out compared with the degree of fracture reduction and screw position [22]. Moreover, a TAD < 20 mm reduced screw cutting out [22]. However, a shorter TAD is not ideal, since shorter TAD and Cal‐TAD often lead to the spiral blade being positioned too close to the cartilage, which greatly increases the risk of spiral blade resection. The tip of the spiral blade should also not be too close to the subchondral bone to avoid damaging nutrition provided by the cartilage. Hsueh et al. have hypothesized that screw resection can be reduced when the lower limit of the TAD of the spiral blade is 15 mm [23]. Conversely, the Born biomechanical test has also shown that an axial cut‐out primarily occurs when the helical blade is implanted too deep [24], and this may be related to the following factors: (1) the helical blade provides percussive implantation during surgery, which fills the bone around the blade to increase its density, and (2) the spiral blade can be implanted too close to the articular cartilage. Regarding the former, the bone at the tip of the blade did not change, while the tip of the helical blade was sharper than the traditional lag screw, making it easy to penetrate.

### 4.3 Reliability and Effects of the TAD and Cal‐TAD

As the distance of TAD and Cal‐TAD increases, the risk of cephalic screw mobilization increases. The general consensus in the literature is that the superior limit of TAD should not exceed 25 mm [19, 25]. However, Caruso et al. have proposed that the TAD value should be relaxed to 30.7 mm [26]. A greater TAD has closely correlated with cut‐out of the device from the femoral head [12]. Nikoloski et al. have proposed that a standard TAD that is < 25 mm is not applicable to PFNA and that a TAD that is < 20 mm has a risk of increasing medial perforation by the spiral blade [19]. Meanwhile, a TAD that is > 30 mm increases the risk of an upward cut‐out. Thus, the range of TAD could be 20–30 mm. Based on our experience, we have found that it is appropriate to maintain TAD and Cal‐TAD in the range of 20–25 mm in PFNA operation of subtrochanteric fractures. Distinct from intertrochanteric fractures, the anatomic and biomechanical characteristics of the subtrochanteric region result in a high concentration of internal stress, and the fracture degree is relatively comminuted. Consequently, the deeper a screw is inserted, the closer it is to the femoral calcar and the stronger the holding force and antirotation ability that is obtained. Therefore, we propose that it is better to implant a screw to a greater depth, albeit without damaging the blood supply of the subchondral bone.

### 4.4 Comparison With Previous Findings

The optimal position of the spiral blade in the femoral head remains a topic of debate. Some scholars believe that the spiral blade should be located in the middle of the femoral neck according to positive and lateral x‐ray films in order to target the intersection of pressure trabecular and tension trabecular bones. At this point, bone density is high, and this supports firm fixation [27]. Meanwhile, other scholars have proposed that anteroposterior and lateral x‐ray films should locate the blade in the middle and lower 1/3 of the femoral neck and in the middle of the femoral neck, respectively. The closer the spiral blade is to the femoral calcar, the greater axial and torsional stiffness can be obtained [28]. Consistent with the results of other studies, our results indicate that the spiral blade should be positioned in Zone V III or Zone V to reduce the incidence of complications. The positions of being forward, backward, upward, or downward often lead to a larger TAD and Cal‐TAD. If the TAD has 20 mm as a boundary, it represents 10 mm in both the anteroposterior and lateral positions. Given that the diameter of the spiral blade is 10 mm, this serves as a reference that can be readily judged visually during surgery to estimate the correct blade position.

### 4.5 Strengths and Limitations

A distinctive feature of this study was its long‐term follow‐up combined with a thorough analysis of postoperative fracture healing rates and the positional distribution of head–neck screws in non‐healing cases. Through rigorous methodology, we have identified the optimal placement of the spiral blade for patients with subtrochanteric fractures and have refined the recommended range for TAD and Cal‐TAD to 20–25 mm. This clarification holds considerable significance for enhancing treatment efficacy and patient outcomes in this specific cohort.

There are several limitations associated with the present study. First, this is a retrospective study. Therefore, prospective randomized controlled trials should be conducted to address the limitations of incomplete data and bias. Second, other factors that can contribute to the failure of subtrochanteric fracture surgery were not considered; these may include lack of medical support, surgical reduction quality, and degree of osteoporosis. To minimize or eliminate the impact of these bias factors on experimental results, future studies should include a larger sample size. Third, the mean age of our consecutive series of patients was relatively young (56.9 years, range: 24–76), and more than half of the cases involved high‐energy trauma (e.g., traffic accidents and falls from height). Consequently, the findings of our study may not be generalizable to lower energy subtrochanteric fractures that occur in elderly patients. In the future, we plan to expand the age range of our sample or specifically focus on studying individuals over the age of 65 years in order to gain a more comprehensive understanding of the research question. Fourth, we have adopted methods such as replacing intramedullary nails, iliac bone grafting, and hip joint replacement as secondary surgeries for patients who do not have a successful result following subtrochanteric fracture surgery performed as described above. However, we do not have a detailed report on outcomes or the revision strategies employed for the non‐union cases examined. Fifth, the clinical trial excluded atypical subtrochanteric fractures caused by long‐term bisphosphonates, which are distinct from fractures caused by osteoporosis or trauma alone. Additional research will be needed to determine whether or to what extent the present conclusions generalize to such atypical fractures. Finally, the groups established represent several controversial subgroups previously reported for intertrochanteric fractures (e.g., ≤ 20 mm, 20–25 mm, ≥ 25 mm). In order to reflect the application value of the two concepts more objectively with respect to the surgical treatment of subtrochanteric fractures, it will be necessary to test multiple segment levels (e.g., 0–5 mm, 5–10 mm, 10–15 mm, 25–30 mm, etc.) and compare the incidence of complications corresponding to TAD and Cal‐TAD for each of these groups.

## 5 Conclusions

The results of this retrospective study indicate that the use of PFNA internal fixation in the treatment of unstable femoral subtrochanteric fractures with placement of the spiral blade in the middle or lower 1/3 of the femoral neck does not increase the incidence of complications. However, having a range of 20–25 mm for the TAD and Cal‐TAD can reduce the incidence of complications.

## Author Contributions

**Qingyan Zhang:** conceptualization, data curation, formal analysis, investigation, methodology, software, validation, writing – original draft. **Xiaogang Wang:** conceptualization, data curation, writing – review and editing. **Longhui Su:** conceptualization, data curation, investigation, writing – review and editing. **Qiang Xu:** data curation, formal analysis, investigation, methodology, project administration, resources, software, supervision, validation, writing – review and editing.

## Ethics Statement

The study protocol was approved by the Ethics Review Board of Sichuan Orthopedic Hospital (approval number: KKY2021–026–01). All procedures were performed in accordance with the Declaration of Helsinki and relevant policies in China.

## Consent

We have obtained written informed consent from all study participants. Written informed consent was obtained from the patients and/or their families to allow data to be submitted for publication.

## Conflicts of Interest

The authors declare no conflicts of interest.

## Data Availability

The datasets generated and analyzed during the current study are available from the corresponding author on reasonable request.
